# Global positioning system: a new opportunity in physical activity measurement

**DOI:** 10.1186/1479-5868-6-73

**Published:** 2009-11-04

**Authors:** Ralph Maddison, Cliona Ni Mhurchu

**Affiliations:** 1 Clinical Trials Research Unit, University of Auckland, Auckland, New Zealand

## Abstract

Accurate measurement of physical activity is a pre-requisite to monitor population physical activity levels and design effective interventions. Global Positioning System (GPS) technology offers potential to improve the measurement of physical activity. This paper 1) reviews the extant literature on the application of GPS to monitor human movement, with a particular emphasis on free-living physical activity, 2) discusses issues associated with GPS use, and 3) provides recommendations for future research. Overall findings show that GPS is a useful tool to augment our understanding of physical activity by providing the context (location) of the activity and used together with Geographical Information Systems can provide some insight into how people interact with the environment. However, no studies have shown that GPS alone is a reliable and valid measure of physical activity.

## Background

Prevalence of physical inactivity (doing very little or no physical activity at work, at home, for transport or in discretionary time) is estimated to be 17% globally, whereas the estimate for insufficient levels of physical activity (< 150 minutes moderate or < 60 minutes of vigorous activity per week) is 40% [1]. Lack of physical activity is associated with an increased risk of ischemic heart disease, type 2 diabetes, colon cancer, depression, and breast cancer [1]. Accurate measurement of physical activity is a pre-requisite to monitor levels of physical activity and design effective interventions.

A major limitation of physical activity research to date has been the lack of objective, practical and inexpensive tools to measure physical activity and energy expenditure on a large scale. Currently, doubly labeled water (DLW) is considered the 'gold standard' for the determination of total energy expenditure, however its usefulness for wide scale population based research is limited by participant burden and its excessive cost. Methods such as direct observation are time consuming and impractical on a large scale. Secondary measures such as heart rate monitors (which provide an indication of exercise intensity), accelerometers (which provide an indication of movement counts), and pedometers (step counters) provide objective assessment of physical activity, but no information regarding the context of the activity such as location, distance traveled, and speed [2].

A new and potentially valuable tool for improving the assessment of physical activity utilizes the Global Positioning System (GPS). Greater understanding of the nature of physical activity (or inactivity) is essential if we are to develop and implement effective interventions. Global positioning system (GPS) technology has the potential to improve our understanding of physical activity by providing location information. Researchers have begun to integrate GPS technology into physical activity-related studies, however because the technology is relatively new, only a handful of such research studies currently exist. The purpose of this paper is to provide a review of the literature on the application of GPS to monitor human movement, with a particular emphasis on free-living physical activity. Specifically, the paper will first describe GPS, review how it has been used to assess human movement, and provide recommendations for future research.

### Review methods

Computer (Ovid Medline, SCOPUS, SPORTdiscus) and manual searches of papers in the English language were conducted. Studies were reviewed from 1990 to January 2009. The following search terms were used, Global Positioning System, GPS, Satellite, Physical Activity, Exercise, Sport, Measurement. We also carried out a citation search of included papers. The aim of this review was to focus on peer-reviewed papers; however grey literature was included if it reported on new or developing GPS technology. Studies with both adult and youth populations were included in this review. Twenty-nine papers were included and a narrative description of findings is presented [see additional file 1].

### What is GPS?

The Global Positioning System (GPS) is currently the only fully functional Global Navigation Satellite System (GNSS). Twenty-four GPS satellites currently orbit Earth and transmit signals to GPS receivers, which determine the location, direction, and speed of the receiver. Since the first experimental satellite was launched in 1978, GPS has become an essential instrument for navigation, and an important tool for land surveying and cartography. GPS also provides a precise time reference, which is used in many applications including scientific study of earthquakes and synchronization of telecommunications networks.

GPS was originally developed by the United States (U.S.) Department of Defense. Because of its military application, the U.S. Department of Defense applied selective error, a deliberate error embedded in the system designed to reduce the risk of hostile forces using the highly precise systems. In 2000, the then-President Clinton announced that he had ordered the U.S. military to stop scrambling signals from its Global Position System (GPS) satellite network, thus making the data available to civilian GPS owners [3].

The position of a GPS receiver is calculated by measuring the distance between itself and three or more GPS satellites. Each satellite is equipped with an atomic clock. When first powered on, GPS devices undergo an initialization period, during which they acquire signals from the satellites, and synchronize the GPS clock with the satellite's atomic clock. GPS devices constantly receive and analyze radio signals from the satellites, calculating precise distance (range) to each satellite being tracked. GPS devices use trilateration, a mathematical technique, to determine user position, speed, and elevation.

GPS is now used in a variety of commercial and research applications such as environmental exposure [4], farming [5], ecology [6], driving assessment [7], estimating travel time [8,9] and more recently in exercise science [10]. Technological improvements have resulted in portable GPS units with adequate memory to store positional data over time, thus offering opportunities for obtaining location information at low cost. Despite the improvement in portability, GPS is not without limitations. GPS devices often fail to record position indoors (particularly in concrete buildings) and under heavy tree canopy and in dense urban areas [11].

Prior to reaching the GPS receiver the satellite signal is influenced by a variety of sources such as atmospheric conditions and local obstructions, which can produce error in the calculated distance to the satellite and in turn the computed speed and position. Differential GPS (dGPS) has been used to address this error, and utilizes stationary receivers placed at known ground locations to compare their fixed positions with the position given by the satellites. Using radio waves, the corrected signals are sent from the fixed receivers via a differential receiver to the GPS receiver [10].

GPS units require a period of initialization when they are first powered on, which is when the GPS acquires the signal from the satellites to obtain positional data. The initialization period varies for each specific GPS make and model, but can range from 15 seconds to five minutes. GPS units have a warm and cold signal acquisition period; a cold start is when the unit is initialized having not been used for some time, whereas a warm start refers to when units have been used recently but have not had significant changes in location (such as leaving a building which was previously entered) [12]. These different periods are important because GPS data may not be logged even when the device is moving, which has implications when interpreting and cleaning the data [12].

### GPS as a tool to measure human movement

In 1997 Schutz and Chambaz [13] suggested that GPS could be used to assess human location. They highlighted the following potential advantages of utilizing GPS: "(1) portable, (light and small size); (2) non-invasive non-obtrusive free living measurements; (3) continuous measurement with 'on line' data obtained in a miniature screen, hence feedback for the subject; (4) free access to the GPS satellites in any part of the world; (5) reasonable cost of the GPS receiver; (6) data could be stored and subsequently retrieved if required; (7) the technique can be used to independently validate measurements of velocity of walking and running by other techniques (such as accelerometry)" (p.339). While many of the potential benefits outlined by Schutz and Chambaz's exist, for some parts of the world (particularly developing nations) satellite coverage is poor resulting in limited GPS availability. However, since the Schutz and Chambaz paper was published, GPS technology has been refined more for use as sport tool, rather than an instrument for measurement of free-living physical activity.

### GPS in the sporting domain

From a human movement perspective a number of studies have applied GPS technology to track people's position and speed and to provide a more detailed understanding of sporting performance, with sports including orienteering [10], cross country skiing [14], Australian Football [15] soccer [16], and Golf [17]. Early research validated dGPS measurements of speed, position, and distance among orienteering athletes and showed that dGPS provided accurate information regarding the route traveled and the athlete's speed when compared to chronometry (measured time) [18]. Larsson and colleagues have conducted a number of studies linking GPS with physiological data to help interpret athletes' performance [18,19]. Specifically, these studies showed that laboratory incremental test data could be compared to field test data and that metabolic gas measurement system data could be matched with similar timestamps in the dGPS data; such that the physiological demands of activity compared could be examined in relation to the athlete's position and speed around an orienteering course.

Other sport studies [17] have integrated heart rate data with GPS measurements and showed that it was possible to observe variations in heart rate according to the location and terrain during a round of golf. More recently, GPS has received attention as a useful tool to track player's movements during games of Australian Football [15], soccer [16], and during high-intensity and intermittent exercise (simulated team sport running circuits) [20]. Taken together, these studies have shown that GPS can be used to accurately track player's position and velocity in real time and provide a useful sport performance tool. However, apart from these findings, these studies provide very little information regarding the application of GPS to assess free-living physical activity.

### GPS to measure human movement in controlled conditions

A number of studies have been conducted to assess the accuracy of GPS to measure various physical activities under controlled conditions. In their original validation study Schutz and Herren [21] compared the velocity of walking, running, and cycling at various speeds around a running track with GPS and chronometry. Nineteen different walking, 22, and 35 cycling speeds were tested. Results showed GPS derived walking and running speed compared well with chronometry) [21]. Since then a number of studies have attempted to validate GPS measurement of speed by measuring an individual's speed with commercially available GPS devices over a specified distance [22-24], chronometry [14-16], and a bicycle speedometer [24]. These studies generally reported high levels of agreement between GPS derived speed and manual chronometry [22]; however Duncan et al. [25] found that the position of GPS units influenced measurement. Specifically, lanyard and waistband mounted GPS devices overestimated distance during walking trials but not during cycling trials.

Also in controlled environments researchers have used GPS to assess biomechanics of walking [26-28]. Researchers examined whether walking speed prediction could be improved using parallel measurements of body accelerations and altitude variation using differential barometry during when walking on an outdoor circuit (1.3 km). Results showed that barometry rather than acceleration improved the prediction of walking speed. The authors concluded that dGPS receivers could be used for measuring and tracking outdoor walking in humans [29]. Others [27] have shown that stride frequency measured with accelerometers and GPS were perfectly correlated. As with the sporting domain, dGPS has been combined with indirect calorimetry to assess the mechanical work associated with walking [28] and showed that GPS (in phase mode) was able to record small body movements during walking.

In summary, studies in controlled conditions have focused primarily on validating GPS as a tool to measure walking or running speeds over measured distances against chronometry. Overall, these studies demonstrate that GPS, especially dGPS can accurately record speed, whether it is in trained athletes [14-16] or more recently with patients with peripheral arterial disease (PAD) [23]. GPS combined with other measurement tools such as accelerometry or indirect calorimetry can improve our understanding of the energy cost and biomechanics of walking in controlled situations. Collectively, these studies indicate that GPS has sufficient accuracy to measure walking and cycling speed under controlled conditions.

### GPS to measure human movement in free-living conditions

In terms of free-living activity, the most promising avenue for the application of GPS is to augment accelerometer-based measurement of physical activity [11,27]. This approach potentially provides greater insight into the nature of activity with both location and intensity information available. Greater understanding of where activity takes place or indeed where people are most sedentary would permit a more targeted approach to implementing physical activity initiatives and interventions.

Rodriguez, Brown, and Troped [11] examined the feasibility of integrating GPS and accelerometer data to assess adult physical activity. Two studies were conducted; the first investigated the battery life, reliability, and validity of commercially available GPS watches (Garmin Foretrex 2001; Garmin Ltd., Olathe, KS), which were WAAS enabled. WAAS refers to Wide Area Augmentation System, which is a system of satellites and ground stations that provide GPS signal corrections to improve position accuracy. On average, battery life was 15.97 hours. Overall, the GPS receivers were found to be reliable and valid measures of location - when compared to a geocoded static location point; the GPS coordinates were on average within 3.02 meters (SD 2.51). The devices inter-unit reliability were tested in various environmental conditions, open space with some tree canopy; clustered development (high-density development with open space around it), and urban. The mean distance difference among units ranged between 10.7 meters (SD 11.9) and 20.1 meters (SD 21.8).

In a second study, thirty-five adult participants wore Garmin Foretrex 201 GPS units and Actigraph accelerometers simultaneously over three consecutive days. Accelerometer-derived physical activity data were classified into bouts of moderate and vigorous intensity, with each bout matched to GPS positional data. Participant's GPS data were overlaid with Geographic Information Systems (GIS) information to determine the location (i.e., indoors, outdoors in the neighborhood, outdoors etc). Results showed GPS data were available for approximately 60% of all activity bouts, of which 46% occurred within the vicinity of the participant's neighborhood. Participants who got most of their moderate and vigorous physical activity in their neighborhood tended to live in environments with higher population density, higher housing unit density, higher street connectively and higher street density. The authors concluded that data recorded using portable GPS devices were sufficiently precise to track individual's movements.

The integration of GPS has more recently been applied to free-living physical activity in adolescents [30] and children [31]. In the first study, high school students (n = 110) wore a Garmin (Garmin Ltd., Olathe, KS) Forerunner 305 GPS watch and an Actigraph accelerometer for four consecutive days. GPS and accelerometer data were integrated with GIS to map each participant's neighborhood to identify the main locations of moderate-vigorous bouts of activity as well as to describe typical activity patterns [30]. Overall results showed that during weekdays, activity was primarily located within the individual's school and neighborhood environments, whereas activity patterns were more disparate on weekend days. These findings mirror that of a U.S. pilot study [32] which found there was much less variability in travel patterns on weekdays with more time spent within the vicinity (one km) of the home. Results showed that adolescents traveled further from home in the evenings and early mornings on the weekends.

The second study titled Project CAPABLE (Children's Activities, Perceptions And Behavior in the Local Environment) [31] combined GPS units (Garmin Foretrex; Garmin Ltd., Olathe, KS) with RT3 triaxial accelerometers and self-report travel diaries in children aged 8-11 years to determine the nature (speed, intensity, and direction) of children's physical activity behaviors in the presence or absence of an adult or other children. Findings indicated that children's travel behaviors differed depending on whether they were accompanied by an adult or not. Approximately 56% of children were allowed to go out on their own, with boys being allowed out more often than girls. Children who were allowed to go out alone were more likely to visit a friend's house after school. Children generally walked slower when accompanied by an adult than when unaccompanied. Moreover, participants walked in a more exploratory way when not accompanied by an adult. The study also showed that walking trips on pavements tended to be more energetic and purposefully on roads than in open spaces. This study presents a novel approach for using GPS and accelerometry with self-reported behavior to assess not only how children interact with their environment, but also how others (adults, peers etc) affect that interaction.

Others have investigated the feasibility of combining GPS and heart rate monitoring to measure physical activity in primary school children (n = 39) [33]. In New Zealand, spatial data were collected using a GPS unit and heart rate receiver during a school lunch break. Results showed that GPS could discriminate the velocity of play-related activity. Heart rate was used to quantify the energy expenditure associated with different movement speeds, such that children who moved at slower speeds expended less energy than those moving at faster speeds.

A component of free-living physical activity is active transport in which people walk or cycle to school or their place of work. A number of research studies have incorporated GPS to assess travel routes in urban environments [34]. Generally such research has shown that GPS can be used to differentiate travel mode such as riding a bicycle or traveling by car [8,35]. Moreover, recent research [36], has found that GPS measured traveled distance compared well with GIS-estimated travel distance. In an Australian study, primary school children (n = 75) carried a Garmin (Garmin Ltd., Olathe, KS) etrex GPS device during the journey to and from school on a single occasion, with home and school addresses reported by parents geocoded in GIS. Children were instructed to travel their normal route to and from school. An interesting finding from this study was that the data from the GPS travel route revealed that students avoided busy streets and intersections, thereby providing interesting insight into how environmental factors can inhibit or possibly promote physical activity. Another GPS study with adults in the U.S. showed that equal amounts of bicycling time took place on roads with and without cycling infrastructure (such as streets with cycle lanes or separate pathways [34]. This study highlighted the utility of GPS to not only assess a physical activity behavior (e.g., bicycling) but how the built environment can have an impact.

Recent research has combined GPS and accelerometry in an attempt to discriminate physical activities (walking, jogging/running, bicycling, and in-line skating) [37]. The combined data (accelerometer counts, steps, and GPS speed) were able to correctly classify the activity mode for 91% of observations. A similar study was conducted [38] to differentiate daily activities and sports performed in supervised and unsupervised settings. Participants wore triaxial accelerometers on their hip and wrist and GPS loggers during 21 hours of supervised and 47 hours of unsupervised activities, which included lying down, sitting and standing, walking, running, cycling with an exercise bike, rowing with a rowing machine, playing football, Nordic walking, and cycling with a regular bike. Activity recognition was conducted using signal features derived from the triaxial accelerometers and GPS information. When both supervised and unsupervised data were considered, the total accuracy of the activity recognition was 89%.

Taken together these studies suggest that GPS is a useful tool to augment our understanding of physical activity by providing the context (location) of the activity and used together with GIS can provide some insight into how people interact with the environment. However, no studies have shown that GPS alone is a reliable and valid measure of physical activity.

### Barriers to using GPS

An obvious problem associated with utilizing GPS with other movement devices to measure physical activity is that two devices are required. To address this, researchers have begun to use other GPS enabled and frequently used technology such as cellular telephones [24,39] and personal digital assistants [34]. However cell phones have only shown adequate reliability to track position and are limited under some conditions such as public transportation [39]. Despite this, the use of cell phones is not without problems. The battery life of GPS enabled cell phones is less than GPS data loggers, people may not choose to carry cell phones during in more intense physical activity, which has adherence implications. Moreover the limited memory capacity of some GPS-enabled phones needs to be overcome before this technology can be widely used to augment physical activity measurement. To reduce the burden of wearing multiple devices, Japanese scientists [40] have developed the jogging support system, which integrates GPS, heart rate monitoring, and accelerometry into clothing; however no validation data are currently available. Others [41] have developed a prototype integrated system which collects and subsequently combines data from an activity monitor and GPS device. Although not directly designed to assess physical activity, Elgethun et al. [42] incorporated GPS instruments into the clothing of eleven young children (2-8 years) to provide time-location data for exposure assessment studies. These GPS devices provided good spatial resolution to locate participants and distinguish various activities. This approach has the potential to overcome many of the difficulties associated with assessment of physical activity in this population (e.g., recall bias, proxy reports and the burden of direct observation). Moreover, wearable technology would facilitate the time-activity-location studies and provide valuable insight into the nature of physical activity among this population.

## Discussion

GPS has been used successfully to augment the measurement of physical activity. Recent research has shown that portable GPS devices are reliable and can be incorporated with accelerometry to supplement physical activity measurement in adult and adolescent populations. GPS has also been used to accurately measure travel routes in children and to differentiate selected physical activities. In the sporting domain, GPS has been used to track athlete movements and complement physiological assessment. Taken together, these studies support the use of GPS as a complementary tool for the measurement of physical activity and human movement, rather than a physical activity measure per se. A major advantage of using GPS is that it provides much needed contextual information (e.g., location) of physical activities, thereby providing a better understanding of how people interact with their environment, whether this is during free-living physical activity, active transportation, or sport and recreation. Some studies [36] have shown that travel routes and physical activity behaviors are influenced by environmental factors such as intersection density, housing density and street connectivity. This research needs to be extended to explore potential associations between the built environment and physical activities.

There are a number of considerations when embarking on GPS research. First, there is no standard approach for the analysis and interpretation of GPS data. Previous research studies that have integrated GPS and GIS have exclusively utilized an individual approach such as the generation of individual maps presenting snail trails of activity [11,30]. Alternative and more sophisticated approaches are required to analyze and interpret data at the group level. For example, GPS coordinates could be pooled to create flow diagrams, which could be used to identify typical travel routes or activity patterns; however this approach would require large sample sizes. While GPS provides the location of activity it is unable to readily distinguish the type of physical activities undertaken. For example in physical activity studies that have produced snail trails, description of activity type was not provided; therefore it was not possible to determine whether periods of very low intensity activity were due to walking slowly or traveling by motorized transport or bicycle. Preliminary research has been conducted [37] to distinguish types of activity using GPS and accelerometry for a limited number of activities. Research examining travel surveys has shown that it is possible to differentiate travel mode such as riding a bicycle or traveling by car [8,35]; however this research needs to be developed further in the physical activity domain and would only improve the utilization of GPS to understand physical activity behavior.

GPS signal capture is affected by environmental conditions (heavy tree cover, being inside buildings; dense urban location), and therefore results in data loss. Managing the loss of coordinate data is an important issue, and one that was poorly described in the reviewed studies. One study [30] replaced missing data using the last value carried forward providing the previous value was within 100 meters of the original. This was an arbitrary value and assumed participants had not moved more than 100 meters. Wiehe et al. [32] also used a data imputation approach. Specifically, when more than 5-minutes but less than one hour had elapsed between data points, interim 5-minute time points were imputed. If two adjacent points bounding a period of missing data were with 30 meters, a last value carried forward was used. For data points more than 30 meters distant data were imputed using the data point closest to home. Future studies should detail management of missing data. There are lessons however to be learned from other fields that have processed GPS data from travel surveys [43], in which data loss may occur during rail travel through tunnels or underground networks or during dense urban areas. To address this researchers have developed algorithms based on GPS data and GIS sources to correctly determine travel routes [12]. Others have developed software that identifies the cause for the signal loss and provides calculations to manage the lost data [43]. Use or adaptation of these approaches should be considered for free-living physical activity research.

There are some important limitations of GPS research to date. The use of two separate devices (e.g., GPS and accelerometer) limits scalability of this approach due to cost and participant inconvenience. As technology improves, it should be possible to incorporate GPS into movement devices such as accelerometers. Moreover, given the pervasive use of cellular telephone technology, the inclusion of movement devices and GPS into cellular phones [39] might improve compliance. While researchers have incorporated technologies such as GPS, accelerometers and heart rate into wearable vests or clothing; this approach still has implications in terms of participant burden and comfort when wearing these items. Portable GPS devices such as the Garmin Foretrex and Forerunner have limited continuous battery life (approximately 11-16 hours) [11,30], and require users to recharge the device overnight. This has implications for individual compliance, particularly among the younger and older aged populations who may not recharge the device.

The publication or presentation of maps which include GPS and GIS data poses threat to participant privacy because of the potential for reverse identification [44]. Two recent papers have shown that it is possible to reverse identify people's actual residences and thereby compromise individual's privacy [45]. The first [44] paper correctly identified 26% of people's addresses using reverse identification methodology using a hypothetical low-resolution map of geocoded addresses. Using mortality data from Hurricane Katrina the second study showed that 66% of mortalities re-engineered from a map location could be identified to actual houses. To avoid compromising people's privacy it is possible to apply masking procedures to the point of placement of human cases on a map. For example, some researchers have randomly reallocated case within a given distance of their true location [46]. A common approach to de-identifying such data is to use ZIP or postal codes rather than home addresses to preserve anonymity [44]. Brownstein et al. [44] highlighted the need for guidelines for the display or publication of health data to guarantee privacy protection. Moreover, editors of journals or books might consider implementing policies to ensure safe reporting of spatial data.

In terms of physical activity, there are many opportunities for future GPS research. The greatest utility for GPS is to help understand how people interact with their environment. For example, future studies could utilize GPS to determine individual's exposure to obesogenic environmental conditions such unhealthy food advertising or outlets. Alternatively, GPS could be used to determine exposure to, or use of physical activity facilities (such as swimming pools, gyms etc.) and determine the association of physical activity and sedentary behaviors with specific locations and environments. Further research is also required to refine techniques to differentiate the type of activity using GPS and accelerometry. Future physical activity research should incorporate spatial analysis to explore activity patterns across time. This approach has been used successful in environmental exposure research [47] and more recently with adolescent girls travel patterns [32]. Using time series GPS data in association with GIS it is possible to determine how physical activity patterns change in terms of proximity to work or home over a day or week. A spatial approach also has potential to assess the effectiveness of physical activity interventions by examining how activity patterns change over time and location.

Finally, although a relatively new technology, there needs to be some consensus or guidelines for the use of GPS in free-living activity research. These guidelines would outline the minimum battery requirements and frequency of data recording as well as management and interpretation of the data. This consensus would build on existing evidence and help established and new researchers to conduct high quality research, while preventing them from making the same trial and error mistakes made by previous researchers. Also, collaborating with researchers from associated areas such as transportation can only advance the field of physical activity-related GPS research.

## Conclusion

In its current form, GPS is a useful complementary tool to augment the understanding of physical activity behavior rather than a stand-alone measure. Future research needs to (1) focus on improving the analysis and interpretation of GPS data; (2) understanding how people interact with their the environment in terms of physical activity; and (3) using GPS as a tool to help evaluate the effectiveness of interventions and as for monitoring changes in physical activity patterns over time.

## Competing interests

The authors declare that they have no competing interests.

## Authors' contributions

RM conducted the review of literature and wrote the manuscript. CNM contributed to the writing of the manuscript. All authors read and approved the final manuscript.

## Supplementary Material

Additional file 1 **Summary of GPS-related human movement studies**. Word document summarizing the GPS-related human movement studies. Click here for file
