# Supplementary material for: Global positioning system: a new opportunity in physical activity measurement
Source: Int J Behav Nutr Phys Act. 2009 Nov 4;6:73. doi: 10.1186/1479-5868-6-73 (PMC2777117; doi:10.1186/1479-5868-6-73)
Supplement: Additional file 1 — Summary of GPS-related human movement studies. Word document summarizing the GPS-related human movement studies. [file 1479-5868-6-73-S1.doc]

**Additional file 1**

| **Author** | **Aim** | **Sample** | **Methods** | **Measures** | **Results** |
| --- | --- | --- | --- | --- | --- |
| Coutts, A. & Duffield, R. 2008 [20] | To assess the validity and intra-model reliability of different GPS devices for high-intensity, intermittent exercise performance. | N= 2; moderately trained male athletes  Mean age = 32 years | Eight bouts of a standard circuit involving intermittent exercise. Six laps around a 128.5 m course. | Six GPS devices (GPS sports Canberra ;  2 x SPI-10, 2 x SPI-Elite, and 2 x WiSPI) tightly packed into a harness together with the antennas clearly exposed.  Battery life not described | Coefficients of variation were good for total distance (3.6-7.1%, and peak speed (2.3-5.8%) but high for high-intensity running (11.2-32.4%) and very high-intensity running (11.5-30.4%) for all GPS devices. |
| Dill, J. 2009 [34] | To provide insight on whether bicycling for everyday travel can help US adults meet the recommended levels of physical activity. Using the GPS system, information was collected on cycling behaviour. | N= 166;  Mean age = 41 years;  55% male, regular cyclists | Participants carried a GPS during all bicycle trips for 7 days. | Specially programmed personal digital assistant (PDA), which could either be placed on the bike’s handlebars or on a back rack. Data logging every 3 seconds. The GPS model and battery life were not described. | Participants averaged 1.6 bicycle one-way trips per day. Sixty percent of the cyclists rode for more than 150 minutes per week during the study and nearly all of the bicycling was for utilitarian purposes, while only 5% were for exercise.  Approximately 50% of bicycling occurred on streets with bicycle lanes, separate paths, or bicycle boulevards. |
| Duncan, M. et al. 2007 [25] | To determine the  test–retest reliability of GPS units for measuring distance traveled during active transport (walking and cycling)  A second aim was to determine whether GPS unit placement influenced accuracy. | N= 19;  Aged 18-33 years | Participants completed two walking and cycling trials at self-selected speeds on a measured 1489-m course wearing two Garmin GPS units, worn in lanyard and waistband placements. GPS estimates of travel distance were compared with actual distance. | Garmin eTrex GPS units worn on lanyard and waistband placements. Manufacturer-reported battery life of 22 hours. Data logging automatic but interval not reported. | Lanyard and waistband units overestimated distance travelled during walking but not cycling. Relative technical error of measurement of raw data ranged from 3.7 to 15.5%. Absolute errors ranged form 5.0 to 8.5%. |
| Duncan, M. & Mummery, K. 2007 [36] | To examine the utility of GPS to measure actual routes taken compared with GIS-estimated travel distance. | N= 59;  Mean age = 11.1 ± 0.79 years | Travel route taken and distance travelled were calculated using GIS shortest route analysis between the student’s home and school. Participants travelled with the GPS during a single journey to and from school. | Garmin etrex GPS unit worn on a lanyard around the neck. Data logging and battery life not described. | GIS provides estimates of travel distance were similar to GPS-measured actual travel distances.  GIS-estimated travel routes crossed a significantly (*p<*0.05) higher number of busy streets (GIS: 1.68±0.12 vs GPS: 1.19±0.11) and traveled on a higher ratio of busy streets to total streets traveled on (GIS: 0.46±0.03 vs GPS: 0.35±0.04) (*p<*0.05) compared with GPS-measured actual travel routes. |
| Duncan, S et al. 2008 [33] | A feasibility study to trial a combination of  GPS surveillance and HR monitoring. | N= 39; 20 boys  Mean age = 8.09 ± 0.56 years | Spatial location and heart rate data were recorded during a school lunch break using an integrated GPS/HR receiver. | 12-channel F500 GPS receiver units were attached to the back of the children using the manufacturer’s harness. Data logging at 1 Hz, and 1 sec intervals. Battery life not described  Polar heart rate receiver with 1 sec data recording | Children averaged a total distance of 1.10±0.56 km at speeds ranging from 0 to 18.6 km/h^−1^. Activity patterns were characterised by short bursts of moderate to high speeds followed by longer periods of slow speeds. In addition, boys averaged higher speeds than girls (1.77±0.62 km/h^−1^ and .36 ±0.50 km/h^−1^, respectively; *p* = 0.003). The percentage of time spent at 0 km/h^−1^ (stationary) ranged from 0.1% to 21.3% with a mean of 6.4±4.6%. |
| Edgecomb, S. & Norton, K. 2006 [15] | To compare the validity and reliability of GPS and computer based tracking systems (CBT) for measuring players distances in Australian football | N= 1. Participant not described | Validity and reliability tests were conducted over pre-determined courses.  For the computer based tracking system (CBT) comparison study, performed 28 trials around different circuits of different lengths and geography with movements tracked with CBT and GPS. | SP 10 GPS Sports System device worn in a harness on the participant’s back. Data logging and battery life not described. | CBT overestimated actual values by an average of 5.8%. The GPS system overestimated actual values by 4.8%. Both showed relatively small errors in true distances. |
| Elgethun, K. et al. 2003 [42] | To (1) develop and pilot test a GPS suitable for studies with children exposed to environmental contaminants; and (2) field study to determine feasibility of using GPS to track children’s movement. | N= 11; 5 male, 6 female  Age, 2-8 years olds (mean = 5.5) | For the field study participants wore custom-designed GPS data loggers integrated into clothing for one entire day. | Purpose built GPS personal acquisition logger units, with battery pack, a central electronic unit, and antenna. GPS units were concealed in closed pockets on the front of the garment. The antenna was placed on the top of the shoulder. Data logging 5 sec with 25 hours battery life using four AAA alkaline batteries. | GPS units provided adequate spatial resolution to locate subjects and distinguish a variety of human activities.  Location could be tracked inside and outside some buildings. Children considered the device acceptable to wear. |
| Ermes, M. et al. 2008 [38] | To examine how well the daily activities and sports performed in unsupervised settings can be recognised compared to supervised settings. | N= 12; 10 males, 2 females  Mean age = 27.1 ± 9.2 years.  Mean BMI 23.8 ± 1.9 | A total of six hours of data were collected per participant during supervised indoor and outdoor activities as well as unsupervised free time.  Activities included lying down, sitting and standing, walking, running, cycling, rowing and playing football. | Garmin etrex Venture GPs device placed in a backpack. Data logging one per 20 sec. Battery life not described.  ADKL202 accelerometers worn on the hip and wrist | The total accuracy of the activity recognition using both supervised and unsupervised data was 89%. Accuracy was 88% using supervised data only and 17% when using non-supervised activity only. |
| Larsson, P & Henricksson-Larsen, K. 2001 [18] | To investigate whether it would be possible to relate dGPS data with physiological variables in a field test. | N= 10;  Mean age = 21.9 years; male orienteers for field test.  One subject performed all validation tests | Validation speed and distance tests were conducted on a measured 115-m straight asphalt road. Participants walked and rand the distance 10 times.  For the field test athletes travelled over a 4.3km orienteering course. | GPS 12CX unit. RXMAR differential receiver. GPS unit placed in a harness and antenna attached to a cap worn on the head. Differential receiver antenna placed around the waist belt of the harness. GPS logging every 5-sec.  MetaMax II metabolic gas analyser | The dGPS gave detailed information about a subject’s speed and position and physiological variables could be related to dGPS data. The correlation between dGPS measured speed and chronometry was r = 0.9995-0.996. The mean errors of distance and position measurement were 0.04-0.7% and 1.94-2.13m, respectively. |
| Larsson, P. et al. 2002 [19] | To determine the physiological responses to orienteering by examining the inter-relationship between dGPS data and metabolic gas analysis. | N= 10; elite orienteers  Mean age = 21.9 ± 3.7 years  Mean height 1.87 m ± 0.08. Mean weight 72.9 kg ± 5.5. | Participants performed an incremental treadmill test and physiological data were collected with blood samples and a gas exchange analyser. Participants then completed an orientation course. | Garmin GPS 12CX unit. RXMAR differential receiver. GPS unit placed in a harness and antenna attached to a cap worn on the head. Differential receiver antenna placed around the waist belt of the harness | Physiological and dGPS data were combined for analysis. A correlation (*r*= -0.64, *p*<0.05) was found between a high anaerobic threshold and few orienteering mistakes. A high threshold of decompensated metabolic acidosis and VO_2 peak_ were related to fast overall time (*r*= -0.70 to -0.72, *p*<0.05 and high running speed (*r*= 0.64 to 0.79, *p*<0.05 and 0.01, respectively) and were the best predictors of performance. |
| Larsson, P & Henricksson-Larsen, K. 2005 [14] | Provide a more detailed analysis of performance in cross-country skiing by combining dGPS with physiological variables. | N= 10; male elite collegiate cross-country skiers  Mean age = 17.9 years; | Skiers participated in a freestyle skiing field test (5.6 km), with dGPS and metabolic gas measurements.  Also performed an incremental treadmill threshold test. Combined dGPS with physiological data to provide detailed information. | Garmin GPS 12CX unit. RXMAR differential receiver. GPS unit placed in a vest and antenna attached to a cap worn on the head. Differential antenna placed around the waist belt on the harness. Data logging every 2sec. Battery life not described. | Combined dGPS and metabolic gas measurements made detailed performance analysis possible.  All treadmill test variables correlated highly with speed. |
| Le Faucheur et al. 2007 [22] | To determine whether a low-cost, commercially available GPS can be used to study outdoor walking of healthy subjects. | Experiment 1 n=6 (32 ± 14 yr, 173 ± 8 cm, 64 ± 11 kg)  Experiment 2 n=10 (23 ± 2 yr, 173 ± 8 cm, 65 ± 10 kg)  Experiment 3 n=14 (22 ± 2 yr, 176 ± 5 cm, 70 ± 6 kg) performed twice a PWP of 2000 m long, including two series of 100, 200, 300, and 400 m, respectively, in a random order (different for each subject). | Three experiments. Experiment 1, participants equipped with the GPS receiver performed a prescribed walking protocol (PWP) of 31 min 30 s, three or four times, on an outdoor athletic track.  Experiment 2, participants equipped with a watch, the GPS receiver and an MP3 player, over which different PWPs was vocally recorded, including both prescribed walking and resting bouts to ensure blinded analysis.  Experiment 3, speed and distance calculated by the GPS receiver during series of 100-400 m walking bouts on an outdoor athletic track were compared with actual speed and distance. | GPS receiver/ logger (Garmin GPS 60), with the European Geostationary Navigation Overlay Service (EGNOS) function enabled placed in backpack. External receiving antenna was placed over the backpack. Data logging 0.5 Hz. Battery life not described. | Raw data were inaccurate, but low and high-pass filtering and artifact processing enabled detection of walking and resting bouts with an accuracy of 89.8% (95% CI, 84.4-93.4).  A manual post-processing methodology improved accuracy to 97.1% (95% CI, 93.5-98.8).  Excellent relationship both between actual and processed distances (R^2^ = 1.000) and between actual and processed speeds (R^2^ = 0.947). |
| Le Faucheur, A. et al. 2008 [23] | To compare non-treadmill methods including GPS with the “gold standard” of treadmill analysis to estimate walking capacity in patients peripheral arterial disease (PAD). | N= 24; patients with Fontaine stage II lower-extremity PAD  Mean age = 57 years; 18 men, 6 women  Mean height 169 cm (164 to 172 cm)  Mean weight 81 kg [(71 to 86 kg] | Participants completed a maximal walking distance (MWD) test on a treadmill test with a 10% grade, the six minute walking test, and an unsupervised outdoor walk for at least 45 minutes using GPS. | Garmin GPS 60 unit with European Geostationary Navigation Overlay Service (EGNOS) function enabled placed in the external pocket of a backpack. External receiving antenna was placed over the backpack. Data logging arte was 0.5  Two batteries provided but battery life not described. | MWD on the treadmill was 184 m (144 to 246 m). Self-reported MWD, Walking Impairment Questionnaire distance score, 6-minute walking test score, and GPS-measured MWD were 300 m (163 to 500 m), 28% (15% to 47%), 405 m (338 to 441 m), and 609 m (283 to 1287 m), respectively.  The correlation between the treadmill MWD and GPS MWD was *r*=0.81 (*p<*0.001). |
| Mackett, R. et al. 2007 [31]. | Primary aim to explore the concept of independent behaviour by children, by examining variation by age, gender and access to open space.  Secondary aims were to determine how independence affects behaviour and whether sex differences existed. | N= 162; 74 boys, 88 girls  Years 8-11 | Children wore GPS and accelerometer devices for 4 consecutive days and completed diaries indicating whether travel was accompanied or unaccompanied and involved parental supervision. | Garmin Foretrex 201 worn on the wrist. Data logging set to record data as frequently as possible but interval not described. Rechargeable batteries and participants charged overnight.  RT3 Triaxial accelerometer at one-minute intervals | From the GPS data, children walked faster, straighter and more intensely on the road compared with when they  were walking in open space (parks, fields, and woods). Open space offered more opportunity to play and meander about.  GPS and accelerometry was used to describe children’s travel patterns.  On the road, adult presence made a significant difference to children’s patterns of movement: faster, straighter and more energetic, whereas differences were not significant for off-road. |
| Maddison et al.2009 [30] | To describe the location and intensity of physical activity among New Zealand adolescents. | N=79; 60% male  Mean age 14.45±1.55 years | GPS and accelerometry data were collected for four consecutive days during free living activities. | Garmin Forerunner 305 GPS device worn on the wrist. Data logging using smart sensor variable rate. Battery life 11.5 hours, devices charged overnight. | Participants performed 74 (SD 35.96) minutes of moderate and 7.5 (8.35) minutes of vigorous PA per day, and had on average 414 (SD 100) minutes (7 hours) of recorded GPS data per day. Most of the time spent in MVPA and bout of MVPA were most likely to occur within a one km radius of their school or 150 meters of their home environment during weekdays. Physical activity patterns were more disparate on weekends with most physical activity taking place outside of the home environment. |
| Makikawa, M. et al. 2004 [40] | To develop a jogging support system which incorporated GPS. | N=1; male  Age 22 years  Height 174 cm  Weight 58 kg | Jogging experiment during which the participant walked, ran, and walked for 10 minutes | Jogging support system with a Jemini Systems Producer Associated Co. GPS sensor connected through RS232C serial interface. Communication speed 4,800 bps.  Battery life 2 hours.  Heart rate sensor and accelerometer built into jogging system. | Example data provided however no specific results provided. |
| Michael, Y. et al.2008 [39] | To evaluate the reliability and validity of GPS-enabled cell phones to track outdoor activity. | Research assistant no further description | Phones were carried around an urban and suburban loop. One research assistant carried two phones at one time. Static and free-living assessment. Ten testing points in the urban loop and nine in the suburban loop. Phones were tested in 36 trips | 6 Motorola i760 cell phones. Data sent to server every 5 sec. Average battery life 18 hours 42 minutes. | Overall, the cell phones recorded position in 62.8% of tests. Position acquisition varied by the environment in which the cell phone was carried, highest in open areas (83.5%) and lowest under cover (37.5%). The ability of the cell phones to track varied by mode of transportation. Tracking performance was adequate when moving by car (46.6%) or on foot (29.9%), but low on public transportation (3.8%). The GPS-enabled cell  phone system showed adequate reliability under typical study conditions to track outdoor activity. |
| Peterson, M.N. et al. 2005 [17] | To assess this total volume and intensity of physical activity during a typical round using GPS combined with other physical activity measures. | N= 60; male golfers of various abilities  Mean age: 24.1 ± 5.8 years | Participants wore GPS units and other physical activity measures during a single 18-hole round of walking golf | Commercial SiRf II-based Bluetooth-capable GPS logging Unit (DeLorme) worn on the back of a golf hat. Data logging every 5 sec. Battery life not described.  Participants also wore a MTI accelerometer, Digi-Walker pedometer, and Polar heart rate monitor. | The integrated mapping environment combined heart rate telemetry and GPS logs, showed that it was possible to examine relative physical exertion throughout golf play. High accuracy digital imagery also enabled the assimilation of logged data from each of the golfers, and sequentially provided the necessary medium to track GPS-derived location coordinates of the study subjects along with time-matched heart rate outputs and accelerometer readings. |
| Pino, J. et al. 2007 [16] | To test real time recording of cinematic and physiological variables of football games using GPS. | N= 4;  professional football players Mean age = 22.5 ± 1.8 years  Mean height 172.0 ± 6,7 cm  Mean weight 70.5 ± 2.8 kg | Data was recorded during two 30’ half practice games | FR WD F 500 GPS device and recording unit worn in a harness on the participant’s back the pack. Heart rate transmitter also worn. GPS data logged every second. Battery life not described. | GPS provided real time tracking of position, distance travelled and speed. |
| Perrin, O. et al. 2000 [29]. | To determine whether walking speed prediction could be improved using parallel measurements of body accelerations and altitude variation using differential barometry conditions. | N=14; nine males  Mean age = 24.6 ± 2.4 years  Mean BMI= 21.7 ±2.5 kg m^2^ | Fourteen subjects walked twice along a 1.3km circuit with substantial slope variations (−17% to +17%), with recordings of body acceleration, altitude variation and walking speed using satellite positioning (dGPS). | Two Leica System 500 double frequency GPS receivers. Differential carrier phase used. Antenna mounted on participant.  Data logging 5 Hz. Battery life not described  Single-chip capacitive accelerometer and two THOMMEN HM30 meteorological stations were used | Acceleration alone failed to predict speed (mean r = 0.4). When altitude variation was added prediction improved (mean r=0.7).  Inter-individual variation was found with the altitude/acceleration – speed relationship; however dGPS can be used for outdoor walking speed measurements. |
| Rodriguez, D. et al. 2005 [11] | To determine the usefulness of complementing accelerometer-based physical activity measurement with GPS. | N=35  60% females aged 23-61 years (mean 32.1)  40% males aged 20-58 years (mean 31.5) | Study 1 involved static and field-testing of GPS units for battery life recharge time, validity, inter-unit reliability and body placement. Collected positional data in three different environments.  Study 2 involved a free-living test in which 35 participants wore an Actigraph accelerometer and GPS device for three consecutive days during all waking hours. | Garmin Foretrex 201 portable GPS units.  Data logged every 30 sec with WAAS enabled. Average battery life 15.97 hours (SD 1.01)  Actigraph accelerometer data collection 1 min epoch | Average distance from each unit to the geodetic point was 3.03 m. Average bias among units using Bland Altman plots was 0.9 m, ranging from -0.22 and 1.86 m. Inter-unit reliability in the built environment contexts was acceptable (mean difference among units ranged between 10.7 and 20.1 m.  GPS were available for 59% of all bouts of which 46% were in the participant’s neighborhood. Most MVPA took place in neighbourhoods with higher population density, housing unit density, street connectivity, and more public parks. |
| Schutz, Y. & Herren, R. 2000 [21] | To explore whether a satellite-based navigation system, DGPS, could accurately assess the speed of running in humans. | N=1; Male  Age = 24 years | Subject wore a portable dGPS unit while walking and running at 27 different speeds | Garmin GPS 12CX unit. RXMAR differential receiver. GPS unit placed in a backpack and antenna attached to a cap worn on the head. Differential antenna placed around the waist belt on the harness. Data logging at 0.5 Hz. Battery life not described. | dGPS provided accurate estimation of speed. Coefficient variation was 1.38% for walking, 0.82% for running, and 0.97% for the whole data. |
| Stopher, P et al. 2007 [9] | To use GPS to assess the accuracy of the Sydney Household Travel Survey. | Subsample (n=70) of households participating in the Household Travel Survey were recruited. 45 (64%) provided complete and partial data | In-vehicle GPS provided for each car and of each sampled household. Other participants in the household that did not use a car or were more likely to use public transport were given a wearable GPS device. GPS data collection on the same day as travel diary. | In-vehicle GPS powered by cigarette lighter and wearable GPS. Devices not described in detail. Battery life of wearable GPS and data logging period not described. | GPS travel routes compared to travel survey. Overall respondents under-reported their travel by 7%. Participants also underreported travel distances and over-report travel-times. |
| Tan, H. et al. 2008 [26] | To combine a very low cost accelerometer and GPS unit in to a single unit to enhance the frequency response of the devices used alone.  A second aim was to compare the low-cost data fusion approach scheme for determining average speed and intrastride variation to a traditional high cost survey GPS. | Participants not described | Experiment 1.  Participant cycled six laps around a 400 m track with two laps at the speed of around 3, 5.5 and 8.5–10 ms  -1, respectively.  Experiment 2.  A runner wore a cycling helmet with the Ublox/IMU unit and the antenna on top as well as the Forerunner 305 and ran on country road at a constant speed running with bouts of acceleration and deceleration. | Four GPS units used  1. High cost dual frequency, high accuracy GPS receiver with a maximum sampling frequency of 20 Hz with an external GPS antenna.  2. Low cost WAAS/EGNOS equipped GPS unit with built in antenna, processing algorithm, data logger and a sampling frequency of 1Hz  3. A Garmin Forerunner 305 GPS watch with a maximum sampling  frequency of 1Hz and with post-processing and filtering on board.  4. A low cost single frequency LEA-4T; U-blox GPS receiver outputting raw pseudo-range data at 10 Hz was used in the first experiment and a GPS/IMU system was designed, built by the authors and used in the second experiment.  The combined GPS/accelerometer unit included the receiver, a modified active helical antenna and a tri-axial accelerometer. The accelerometer data were sampled at 250 Hz. | The low cost unit achieved an accuracy of 0.15 ms^-1^ (SD) for horizontal speed in cycling and human running across a speed range of 3–10 ms^-1^. The stride frequency and vertical displacement calculated based on measurements  from the low cost GPS/IMU units had an SD of 0.08 Hz and 0.02m respectively, compared to measurements from high performance  OEM4 GPS units. |
| Terrier, P. et al. 2000 [27]. | To determine whether different gait parameters could be recorded by using commercially available GPS units. | N= 8; 50% males,  Mean age = 26.5 ± 1.8 years | Participants walked 5 different runs on an outdoor track, which were regulated with an electronic metronome | Leica System 500 GPS logger placed in a rucksack tightly attached to the person.  Receiving antenna placed just over the head of the subject, was mounted on a metal bar "fixed onto the dorsal pack.  Data logging at 5 Hz. Battery-life not described.  Physilog triaxial accelerometer | A perfect correlation between average step duration measured by accelerometer and by GPS (*r*=0.9998) was found.  For an average walking speed of 4.0 km/h, average vertical lift and velocity variation were, respectively, 4.8 cm and 0.60 km/h. The average intra-individual step-to-step variability at a constant speed, which includes GPS errors and the biological gait style variation, were found to be 24.5% (coefficient of variation) for vertical lift and 44.5% for velocity variation. |
| Terrier, P. et al. 2001 [28] | To illustrate the application of GPS in the field of locomotion studies. | N=5; three men  Mean age, 26.0 ± 2.1 years  Mean BMI, 21.6 ± 0.9 kg·m^-2^. | Participants walked five times for 5 minutes on an outdoor track at different stride frequencies.  Two minute break between walks. Electronic metronome helped to regulate stride frequencies  Walking speeds ranged from 2.8 to 6.30 km·h^-1^  A portable indirect calorimeter recorded breath-by-breath energy expenditure. | Two Leica System 500 double frequency GPS receivers in backpack. Antenna, placed on head. Differential carrier phase localization was used. Data logging at 5 Hz  Breath-by-breath pulmonary gas exchange system was used (K4b^2^, Cosmed) | For a walking speed of 1.05 ± 0.11 m·s^-1^, the vertical lift of the trunk (43 ± 14 mm) induced a power of 46.0 ± 20.4 W. The average speed variation per step (0.15 ± 0.03 m·s^-1^) produced a kinetic power of 16.9 ± 7.2 W.  Compared with commonly admitted values, the energy exchange (recovery) between the two energy components was low (39.1 ± 10.0%), which induced an overestimated mechanical power (38.9 ± 18.3 W or 0.60 W·kg^-1^ body mass) and a high net mechanical efficiency (26.9 ± 5.8%).  GPS (in phase mode) is able to record small body movements during human locomotion. |
| Troped, P. et al. 2008 [37]. | To assess how well the combination of GPS and accelerometer data predicted different activity modes – a pilot study. | N= 10; 7 males mean age = 39.0 years), 3 females (mean age = 31.0 years) | Participants wore a GPS unit and accelerometer simultaneously during specified activities, such as walking, running, bicycling, skating and driving a car. A total of 29 bouts were performed by 10 participants | GeoStats Wearable GeoLogger unit worn on a small backpack with the GPS patch antenna fastened to one of the shoulder straps. Data logging every second. Rechargeable battery but battery life not described  Actigraph accelerometer worn on the right hip. | Accelerometry resulted in 90% of activities being correctly classified. The addition of GPS to accelerometry resulted in 93% of all activities being classified. |
| Wiehe, S. et al. 2008 [32]. | Pilot feasibility study to demonstrate the use of GPS in adolescent health-related behaviours and to demonstrate patterns and variability of activity by time of day. | N= 15; females  Aged 14-17 years | Participants carried a GPS enabled cell-phone for 7 consecutive days | Blackberry 7520 cell-phone with integrated assisted GPS.  Data logging every 5 minutes  Battery life not described | Overall participants spent one third of their time more than 1 km from home.  Adolescents travelled further from home in the evenings and early mornings on weekend days (up to 28 km).  On weekdays there was less variability with greater time spent within 1 km from their home. |
| Witte, T. & Wilson, A. 2005 [24] | To assess non-differential GPS as a means of determining speed under free-living conditions. | N=1 cyclist  No other descriptive information | A bicycle was ridden around a running track and a custom-made bicycle speedometer was calibrated. Cycled four laps each at five speeds. Also cycled on a straight road. GPS speed was compared to speedometer speed. | GPS receiver (REB 2100 GPS engine consisting of SiRF star II chipset) worn on the head and connected via serial cable to a laptop computer in a backpack.  Data logging 1 sec  Battery life not described | GPS determined speed was within 0.2 ms^-1^ of the true speed measured for 45% of the true values with a further 19% lying with 0.4 ms^-1^ |
